# CT Brain Utilization After Low-Risk Head Injury in Adults: A Retrospective Cohort Study and Literature Review

**DOI:** 10.7759/cureus.101750

**Published:** 2026-01-17

**Authors:** Yahya Al Fathil, Shees Salman, Mani Suleiman, Fergus Morrison, Orla Maguire, Joe Anthony Rotella

**Affiliations:** 1 Emergency, Northern Hospital Epping, Melbourne, AUS; 2 General Medicine, Northern Hospital Epping, Melbourne, AUS; 3 Data Services, Northern Hospital Epping, Melbourne, AUS; 4 Emergency Medicine and toxicology, The University of Melbourne, Victoria, AUS

**Keywords:** brain ct scan, early ct brain, elderly patients, elderly trauma, emergency medical service, neuroradiology

## Abstract

Introduction

Falls in adults aged ≥65 years contribute substantially to emergency department presentations and mild traumatic brain injury, with variable risk of intracranial hemorrhage. Existing CT decision tools often exclude older adults, resulting in potentially unnecessary imaging. This study aimed to identify predictors of intracranial pathology in all adults and compare older versus younger patients to inform a future prospective study.

Materials and methods

In preparation for a prospective study, we performed a retrospective imaging-selected cohort study analyzing all CT brain studies performed for suspected head injury between June and August 2024. Patients under 18 years and those undergoing imaging for non-traumatic indications were excluded. Clinical variables, including GCS, premorbid function, and anticoagulation status, were extracted for analysis. Logistic regression was employed to assess associations with intracranial pathology, with odds ratios and 95% CIs calculated, and statistical significance determined using a Wald test (p < 0.05).

Results

A total of 110 patients were included in this study, with a median age of 73.5 years (IQR 43.5-82.0). Positive CT brain findings were identified in 9 (8.2%) patients. Anticoagulation status demonstrated a statistically significant association with positive CT findings (p = 0.047).

Conclusions

In adults presenting after a fall, anticoagulation, older age, and new neurological deficits emerged as potential indicators of intracranial pathology, while vomiting and suspected skull fracture showed weaker associations. The small number of CT-positive cases and imaging-selected cohort limit precision and preclude assessment of decision-rule performance. These hypothesis-generating findings will inform a prospective study and the development of a predictive framework to guide safer, more targeted cranial imaging in older adults.

## Introduction

Emergency departments (EDs) worldwide have experienced a sustained increase in patient presentations, resulting in prolonged waiting times, increased use of diagnostic tests, and growing challenges with bed availability and hospital capacity. These pressures have persisted and, in most settings, intensified in the post-pandemic period [1-4].

Falls in patients aged ≥65 years are a common reason for ED presentation, accounting for 20% of presentations and contributing to 14% of hospital admissions in this population [5,6]. In independent older adults, a fall is a strong predictor of subsequent functional decline and discharge to residential care following hospital admission [5,7].

The widespread availability of CT imaging has led to a substantial increase in its use among older adults, partly driven by concerns over morbidity and mortality secondary to delayed or missed diagnoses of significant pathology [8].

Traumatic brain injury (TBI) is defined as an alteration in brain function, or other evidence of structural abnormalities, caused by an external force [9,10]. This can be categorized as mild, moderate, and severe [9]. Mild TBI (mTBI) will be the focus of this paper, which can be broadly categorized as a Glasgow Coma Scale (GCS) score of 15 to 13 [11].

Prior studies have reported that the vast majority of TBI presentations were mild in the populations studied, with 94% of presentations reported from the mTBI cohort [12]. mTBI may also vary in other clinical aspects, influencing the clinician’s pretest probability of intracranial injury and the consequent need for imaging. Systematic analysis of patients aged ≥ 65 years who had presented to EDs following a fall had demonstrated an intracranial bleed rate of 5% [13]. However, this rate reflects the full spectrum of TBI, including cases without direct head impact, and is influenced by the inclusion of mostly lower-quality studies with considerable methodological heterogeneity [13].

mTBI has historically been associated with intracranial hemorrhage (ICH) in approximately 10% of cases, with 3.5% of these patients eventually requiring neurosurgical intervention [14-16]. Variable mTBI definitions in the literature, however, may alter reported pathology rates within this TBI subset [17,18]. Further reporting from the Lancet Neurology commission found that 5% of patients suspected of having mTBI demonstrate abnormalities on CT brain imaging [10,19-21], highlighting the wide range of reported rates of mTBI outcomes.

Given the high burden of mTBI presentations [12], several decision tools have been developed to safely limit the amount of cranial imaging required in these patients [22,23]. The Canadian CT Head Rule (CCHR) is a widely adopted clinical decision tool for assessing the need for CT brain imaging in mTBI [22,24-26].

Further scoring tools are available, such as the New Orleans Rule and the NEXUS Head CT Instrument [23,27]. These have been externally validated; however, they have been shown to have lower specificity than the CCHR [24,25]. A shared theme across these widely used clinical decision rules is the exclusion of patients aged ≥65 and ≥60 in the New Orleans rule, respectively [22,23,27]. With all older adults deemed to require imaging in mTBI regardless of symptoms or mechanism, the additional cost of prolonged ED stays necessitates consideration, with the added burden to already crowded EDs.

It is well established that extended ED stays are associated with delirium in older patients [13,28]. As such, identifying factors that minimize unnecessary imaging in this patient cohort aligns with both optimizing care for older adults and addressing healthcare system demands. In anticipation of a future prospective study to examine this issue, we undertook a literature review and a retrospective cohort study of our ED population to develop a local procedure to safely reduce the volume of cranial CT imaging performed.

This study aimed to identify factors associated with clinically significant intracranial injury in all adults presenting to the ED after a fall, with a secondary aim of subgroup analysis to inform a future prospective study.

Literature review

Current Imaging Guidance

Current guidelines worldwide for the management of mTBI in adults emphasize a high-sensitivity approach to limit missed intracranial pathology while reducing radiation exposure in the under-65 population [29-31].

In 2023, the American College of Emergency Clinicians issued a level A recommendation to incorporate the Canadian CT brain rule into the assessment of mTBI. This was preferred over other decision rules, such as the New Orleans and NEXUS, owing to the lower specificity of these tools compared with the Canadian CT brain rule, leading to more overall imaging [31].

In 2023, the National Institute for Health and Care Excellence (NICE) in the United Kingdom issued updated recommendations for the management of TBI. For cases of mTBI, a GCS score below 15 at two hours post-injury, the presence of a focal neurological deficit, multiple episodes of vomiting, suspected open or depressed skull fracture, or post-traumatic seizure necessitate CT brain imaging within one hour of presentation to investigate for intracranial pathology [30].

Further differentiation in the NICE guidance for adults is given to the patient subset who have sustained some loss of consciousness or amnesia since the injury. Within this population, the presence of any of the following factors necessitates timely CT brain imaging: age 65 or older, bleeding/clotting disorders, or a dangerous mechanism of injury alongside more than 30 minutes of retrograde amnesia of events immediately before the head injury [30]. The 2023 NICE guidelines recommend that patients with head trauma who are receiving anticoagulation, even in the absence of additional risk factors, undergo CT brain imaging within eight hours post-injury. However, if the patient presents beyond this window, the recommendation is to perform cranial imaging within one hour of presentation [30].

New South Wales published guidance on the management of closed head injuries in adults in 2011, which is pending further update [32]. Recommendations for imaging in mTBI are similar to those of the CCHR, with patients aged 65 years or older considered high risk and requiring timely imaging [22,32].

Victorian Trauma Service guidelines (2014) did not stratify mTBI but mandated CT for TBI if any of the following were present: post-resuscitation GCS <9; neurological deterioration (ΔGCS ≥2 or hemiparesis); persistent altered consciousness (GCS 9-13 >2 hours); persistent headache or recurrent vomiting; focal deficits (pupillary asymmetry/reactivity changes or unilateral weakness); suspected/confirmed skull fracture or penetrating injury; and in high-risk patients (age >50, seizures, chronic liver disease, anticoagulation), irrespective of clinical examination [33].

In summary, international mTBI guidelines uniformly regard age ≥65 as an independent high‐risk factor necessitating prompt CT brain imaging, irrespective of injury mechanism or neurological status.

Canadian CT brain rule validation

The CCHR has been extensively validated across multiple studies to assess its diagnostic accuracy in identifying clinically significant intracranial injuries in patients with mTBI [24,34-36]. Published in 2001, the rule was designed to optimize CT utilization while maintaining high sensitivity for detecting injuries requiring neurosurgical intervention [22]. A landmark external validation study comparing CCHR with the New Orleans Criteria (NOC) demonstrated that CCHR achieved a sensitivity of 98.4% and a specificity of 49.6% for detecting traumatic CT findings, reinforcing its reliability in safely limiting CT head imaging in mTBI [24]. Despite its high sensitivity, implementation studies have highlighted variability in clinicians' adherence, influenced by factors such as risk perception, patient expectations, and medico-legal concerns [37].

The rule has been widely adopted in ED guidelines worldwide, as discussed above. However, challenges remain in balancing sensitivity with specificity, as exclusion of patients over 65 prolonging their stay in the ED for diagnostic imaging has been associated with increased rates of delirium in this age group [38-40].

Older Adult Specific Outcomes

Head injury population data from the Australian Institute of Health in 2021 reported that patients aged ≥65 years made up 73% of all head injury-related deaths, with males having the highest rate of 52 head injury-related deaths per 100,000 [41]. This rate was similar to that of patients aged ≥75 in the United States, where they had reported a rate of 54 head injury-related deaths per 100,000 in 2017 [42].

The rates of head injury-related deaths have been demonstrated to be significantly higher in patients aged ≥ 65 in comparison to younger cohorts 41. This, however, compromises the full range of TBI, which is inherently dissimilar to mTBI, which makes up the vast majority of presentations of TBI to EDs [12].

A recent systematic review and meta-analysis of repeat CT brain in mTBI demonstrated similar clinical outcomes with selective imaging protocols compared with routine protocols following the index CT brain [43]. Seven high-risk factors were identified as predictors of clinical deterioration. The presence of ICH volume greater than 10 mL, along with multiple lesions, was the strongest risk factor for clinical deterioration (odds ratio (OR) of 20 and 11, respectively). Age ≥65 was also demonstrated to be a predictor for clinical deterioration with an OR of 4 [43]. Notably, in these higher-risk categories of age ≥65 alongside anticoagulation, the overall neurosurgical intervention rate remained low [43]. The majority of the studies included in this meta-analysis were retrospective; as such, this introduces potential selection and reporting bias [43].

Older Adult Specific Scoring Tools

Regarding safe reduction in the amount of cranial imaging performed in older adults, several specific scoring tools have been proposed in the literature. Brown et al. proposed a risk score to guide CT brain imaging in older adults admitted with falls and confusion [44]. They conducted a literature review utilizing searches in two medical databases, supplemented by hand searching. It was not made clear whether independent reviewers were involved in this search and screening.

From this search, they had identified twelve factors that were associated with significant findings on CT brain imaging in the context of head trauma. All factors were denoted a score of +1, excluding new focal neurological signs (+3) and preceding dementia (-1) [44]. Following this, an audit was performed on consecutive adults admitted to a single medical admission unit. Sixty-six patients who had non-contrast CT brain imaging performed were included, with a mean age of 74.8 years (standard deviation: 12, range: 42-97 years). Scoring >3 on this tool demonstrated a sensitivity of 83%, specificity of 89%, positive predictive value of 63%, and negative predictive value of 96% for identifying a positive imaging study. Positive imaging in this study was defined as any intracranial blood, new ischemic lesions, or any space-occupying lesion identified on the radiology report [44].

Derived from a retrospective single-center cohort without independent imaging review, this scoring system lacks robust evidence for widespread clinical implementation. It has not been externally validated nor subject to subsequent author-led studies since its initial report.

De Wit et al. published a decision tool to help exclude intracranial bleeds without CT imaging in older adults who have fallen [45]. This was a prospective cohort study that enrolled 4308 patients aged ≥65 years at 11 sites in Canada and the United States. Patients enrolled had presented to an ED within 48 hours of a fall, including those who had not sustained a head strike. The mechanism of injury was limited to falls from standing height, from a chair or toilet, and from the bed.

Patients in this study were followed up for 42 days post their initial visit to the ED, with the primary outcome of “clinically important intracranial bleeding” diagnosed within this period 45. This bleeding was defined as bleeding within the cranial vault requiring medical or surgical intervention within 90 days, alongside death within these 90 days [45].

Half of the study population had a clear history of head injury with a median age of 83 (interquartile range (IQR): 75-89). 139 patients were demonstrated to have a clinically important intracranial bleed (3.2%), with 6% of these patients requiring neurosurgical intervention. From this data, a focused falls decision rule was developed with a sensitivity of 95.0% (95% CI 90.0-97.5%) and specificity of 38.0% (95% CI 36.6-39.5%) for the detection of clinically important intracranial bleeding, as defined above [45].

De Wit et al. evaluated two related clinical decision tools to guide CT brain imaging in older adults presenting after a fall. The Falls Decision Rule incorporates four criteria: absence of head impact based on patient or witness history, no new abnormalities on neurological examination, preserved memory of the fall event, and a Clinical Frailty Scale (CFS) score <5. In contrast, the Focused Falls Decision Rule uses a simplified two-item approach. Under this rule, patients who did not sustain a head impact and have no new neurological deficits on examination do not require CT brain imaging [45-49].

This tool has been externally validated in a single-center prospective cohort study, including 800 consecutive patients aged ≥ 65 years presenting within 48 hours of a ground-level fall [50]. This validation study demonstrated 97.9% sensitivity (95% CI 89.1-99.9), 31.9% specificity (95% CI 28.6-35.4), and 99.5% negative predictive value (95% CI 97.1-99.9) for the falls decision rule. Limitations of this validation study include the single-center, level-1 trauma center design, which may limit the generalizability of the results. Clinician‐driven neuroimaging decisions are a further limitation of the rule and subsequent validation, potentially introducing selection bias and overestimating associations between predictor variables and ICH.

Serum biomarkers

Multiple biomarkers have been proposed in the literature in recent years to reduce the number of CT scans performed in patients with mTBI [51-53].

S100 calcium-binding protein B (S100B) is extensively studied as a potential biomarker for detecting radiological abnormalities on CT imaging in patients with mTBI [52-58]. Its utility is primarily linked to its role in reflecting blood-brain barrier (BBB) disruption and astroglial activation following cerebral injury [52,59,60]. In normal conditions, the majority of S100B production is done by astrocytes and does not cross the BBB [52,59]. Following intracranial injury, S100B is released from disrupted glial cells, enabling its measurement in serum [52,60]. The mechanism underlying this diffusion is an area of ongoing research, with proposals of diffusion through a disrupted BBB and a potential role of the glymphatic system in the release of S100B from serum [52,61,62].

Important considerations for the use of S100B include extracranial production, elevation following exercise, variation with skin pigmentation, and an association with neurodegenerative diseases [52,63-65].

Numerous studies have evaluated its sensitivity and specificity in predicting intracranial pathology, with varying thresholds proposed to optimize diagnostic accuracy and minimize unnecessary imaging [57,58,66]. When measured within six hours of injury, the sensitivity and negative predictive value of S100B levels in detecting traumatic intracranial lesions in adults are 97-100% and 92-100%, respectively [56,67-69].

Several studies have compared S100B with other serum biomarkers, such as glial fibrillary acidic protein (GFAP) and ubiquitin C-terminal hydrolase-L1 (UCH-L1), in the assessment of TBI [56,70]. A recently published systematic review and meta-analysis suggests that GFAP and UCH-L1 may offer greater specificity for detecting intracranial lesions than S100B, particularly in mTBI cases [71]. Wichmann et al. performed a prospective cohort study of 379 adult trauma patients, with results of GFAP/UCH-L1 demonstrating 100% sensitivity across multiple patient groups, whereas S100B had slightly lower sensitivity (97.5%) in the unselected trauma cohort [56].

In summary, multiple biomarkers have been proposed and prospectively validated in cohort studies of mTBI management, particularly S100B [57,58]. Emerging evidence suggests that integrating GFAP and UCH-L1 into diagnostic protocols is supported [56]. Further research is required to determine the optimal blood marker in the management of mTBI in older adults.

## Materials and methods

Study setting and population

We conducted a retrospective cohort study at the ED of the Northern Hospital in Melbourne, Victoria. This is the busiest ED in the state, with over 112,000 patients admitted annually [72]. This study used an imaging-selected cohort, comprising adults who underwent CT brain imaging for clinically suspected head injury between June and August 2024.

The primary analytic objective was to describe CT findings and identify clinical characteristics associated with clinically significant intracranial injury among imaged patients, rather than to assess diagnostic performance or imaging avoidance strategies.

We excluded patients under 18 years, those without clinical suspicion of trauma, and scans for non-traumatic indications, such as altered mental status of unknown etiology, stroke syndromes, seizure activity, transient ischemic attacks, suspected intracranial infection or neoplasm, syncope of unclear cause, and systemic conditions affecting cerebral function. CT scans performed on patients who were no longer in the ED at the time of imaging were excluded. Cases with indeterminate CT indications at data extraction were also omitted.

Data collection

Data were collected by collating all requested CT brain studies from the ED during the above period. Following this, each encounter was manually reviewed and assessed through our health services electronic database. Each radiological report verified by a consultant radiologist was included.

Positive imaging findings were defined as acute intracranial abnormalities demonstrated on CT brain imaging. Acute intracranial abnormalities included subdural and extradural hematomas, traumatic subarachnoid hemorrhages, intraparenchymal hemorrhage, and cerebral contusion. Isolated skull fractures were included as positive in this study. Extracranial injuries such as scalp hematomas alongside facial bone fractures were deemed negative in the context of our data collection.

Demographic data of each patient’s age, gender, and transport method to the ED were included. The clinical interaction's account type was recorded. The triage category of each patient was extracted.

The lowest GCS score recorded during each ED presentation was extracted. Medical records were reviewed to identify documentation of a baseline GCS <15 and pre-existing cognitive impairment, including dementia. Premorbid functional status was assessed using the CFS [73]. CFS scores were assigned retrospectively by the study authors through electronic medical record review. Authors were not blinded to CT outcomes, and inter-rater reliability was not assessed.

Regarding the presentation's history, several key clinical factors were extracted from the included literature review. The presence of a “dangerous mechanism” was defined as a pedestrian being hit by a vehicle, ejection from a vehicle, or a fall from greater than one meter or greater than five stairs. These criteria were adopted from the CCHR [22]. Loss of consciousness was recorded alongside retrograde and anterograde amnesia, including memory loss preceding impact. The presence of headache was documented, along with recurrent vomiting exceeding two episodes, given its potential association with raised intracranial pressure and underlying intracranial pathology [74]. The anticoagulation status of each patient was assessed, along with the presence of antiplatelet therapy.

Clinical assessment data points included documentation of confusion observed at the time of ED evaluation. The clinician's suspicion of skull fracture based on examination findings was recorded, along with any newly identified neurological deficits. Clinical suspicion of head injury was defined by documentation in ED notes or CT requests indicating trauma, including head impact, loss of consciousness, amnesia, or neurological symptoms. Patient records with unclear or non-trauma-related presentations and imaging indications were excluded to ensure only CTs performed for suspected head injury were included.

Objectives

The primary objective of this study was to identify clinical factors associated with clinically significant intracranial injury in adults presenting to the ED following a fall. The secondary objective was to compare patients aged ≥65 years with younger adults to identify factors more strongly associated with acute intracranial abnormalities in the older cohort.

Analysis

Logistic regression was used to evaluate associations between these clinical features and intracranial pathology, yielding ORs with 95% CIs to quantify risk. Candidate predictors were selected a priori based on clinical relevance and prior literature. Missing predictor data were handled using complete-case analysis. Statistical significance was assessed using Wald tests with a two-sided p-value <0.05. All analyses were performed using R version 4.3.1 (The R Foundation for Statistical Computing, Vienna, Austria).

Ethical consideration

Ethical approval for this study was granted on December 27, 2024, by the Research Development and Governance Unit of Northern Health (approval number: 73.2024). Given that the study was conducted as an audit of imaging practices and outcomes within a single center, patient consent was not required, as data collection involved retrospective review of existing records without direct patient interaction or intervention.

## Results

Of 760 patients who underwent CT brain imaging during the study period, 110 met the inclusion criteria. The median age was 73.5 years (IQR 43.5-82.0). Most presentations were of moderate to high acuity (Australasian triage category 3: 58.2%; category 2: 31.8%). The median lowest GCS score was 15 (IQR 14-15), and 15.5% presented with a GCS <15. Dementia was documented in 11.8% of patients. The median CFS score was 4.0 (IQR 1.0-6.0), with 3.6% missing data. Most patients had heart rates between 60 and 100 bpm (86.2%) and systolic blood pressures between 121 and 160 mmHg (62.4%). Further characterization is displayed in Table 1.

**Table 1:** Demographics of study population IQR: interquartile range, LOC: loss of consciousness, ED: emergency department, GCS: Glasgow Coma Scale, HR: heart rate, BP: blood pressure, DOAC: direct oral anticoagulant

	Negative	Positive	p	Missing (n)
n	101	9		
Gender = female (%)	45 (44.6)	4 (44.4)	1.000	0
Age (median (IQR))	73.00 (41.00, 82.00)	80.00 (78.00, 87.00)	0.116	0
Transport to ED = private (%)	27 (26.7)	5 (55.6)	0.118	0
Triage category (%)			1.000	0
1	1 (1.0)	0 (0.0)		
2	32 (31.7)	3 (33.3)		
3	59 (58.4)	5 (55.6)		
4	9 (8.9)	1 (11.1)		
Lowest GCS (median (IQR))	15.00 (14.00, 15.00)	14.00 (14.00, 15.00)	0.119	0
Baseline GCS less than 15 = yes (%)	14 (13.9)	3 (33.3)	0.143	0
Documented dementia = yes (%)	11 (10.9)	2 (22.2)	0.288	0
CFS score (median (IQR))	4.00 (1.00, 6.00)	5.00 (3.00, 6.00)	0.572	4
Lowest HR recorded (%)			1.000	1
>100	6 (6.0)	0 (0.0)		
60-100	85 (85.0)	9 (100.0)		
Less than 60	9 (9.0)	0 (0.0)		
Highest systolic BP recorded (%)			0.718	1
100-120	20 (20.0)	3 (33.3)		
121-160	62 (62.0)	6 (66.7)		
161-180	11 (11.0)	0 (0.0)		
181-200	7 (7.0)	0 (0.0)		
Dangerous mechanism = yes (%)	1 (1.0)	1 (11.1)	0.158	0
LOC = yes (%)	24 (23.8)	1 (11.1)	0.681	0
Amnesia = yes (%)	16 (15.8)	2 (22.2)	0.639	0
Amnesia of events before impact = yes (%)	10 (9.9)	0 (0.0)	1.000	0
Confusion at time of review in ED = yes (%)	24 (23.8)	3 (33.3)	0.686	0
Headache = yes (%)	21 (20.8)	1 (11.1)	0.684	0
Vomiting >2 episodes = yes (%)	3 (3.0)	1 (11.1)	0.293	0
Suspicion for skull fracture on clinical exam = yes (%)	15 (14.9)	2 (22.2)	0.627	0
New neurology = yes (%)	1 (1.0)	1 (11.1)	0.158	0
Anticoagulated (%)			0.047	0
DOAC	18 (17.8)	5 (55.6)		
No	82 (81.2)	4 (44.4)		
Warfarin	1 (1.0)	0 (0.0)		
Aspirin or other anti-platelet (%)			1.000	0
Aspirin	12 (11.9)	1 (11.1)		
Clopidogrel	4 (4.0)	0 (0.0)		
No	85 (84.2)	8 (88.9)		

Positive CT findings were observed in nine (8.2%) patients, including acute intracranial hematomas in six (5.5%). One was crossing the midline, and one had a midline shift present with a further subdural hematoma.

Among the 110 patients, 72 (65.5%) were aged 65 years or older, while 38 (34.5%) were aged 65 years or younger. Further analysis is included in Table 2. The age of CT-positive patients was 80 years (IQR 78-87), vs. 73 years (IQR 41-82) in negative cases. Median GCS was 14 (IQR 14-15) versus 15 in CT-negative cases (p = 0.119). Five (55.6%) of CT-positive patients were on direct oral anticoagulants (DOACs), compared to eighteen (17.8%) in CT-negative cases (p = 0.047).

**Table 2:** Subgroup analysis of older adults against adults under 65 years of age GCS: Glasgow Coma Scale, CFS: Clinical Frailty Scale, IQR: interquartile range, SBP: systolic blood pressure, CT: computed tomography

	>65 years (n = 72, 65.5%)	≤65 years (n = 38, 34.5%)	p-value
Triage acuity	Category 2: 28 (38.9%)	Category 3: 26 (68.4%)	0.041
Baseline GCS <15	14 (19.4%)	3 (7.9%)	0.09
Documented dementia	13 (18.1%)	0 (0%)	–
CFS median (IQR)	5 (3-6)	1 (1-2)	<0.001
Bradycardia (<60 bpm)	8 (11.1%)	1 (2.6%)	0.09
Hypertension (SBP >160 mmHg)	15 (21.4%)	3 (7.9%)	0.04
Loss of consciousness	13 (18.1%)	11 (28.9%)	0.14
≥2 vomiting episodes	5 (6.9%)	0 (0%)	0.03
Suspected skull fracture	15 (20.8%)	3 (7.9%)	0.06
Acute CT-positive findings	8 (11.1%)	1 (2.6%)	0.09
Neurosurgical intervention	0 (0%)	0 (0%)	–

Logistic regression analysis

Given the small number of CT-positive events, the regression analyses were exploratory. Anticoagulation with DOACs was associated with increased odds of intracranial pathology (OR 5.7, 95% CI 1.4-25.1; p = 0.016). Older age (OR 1.02; p = 0.219) and baseline GCS <15 (OR 3.11; p = 0.138) showed non-significant trends toward higher risk. Dementia (OR 2.34; p = 0.325) and CFS score (OR 1.09; p = 0.534) were not associated with CT positivity (Table 3).

**Table 3:** Multivariable logistic regression analysis of predictors for positive CT findings CT: computed tomography, ED: emergency department, GCS: Glasgow Coma Scale, CFS: Clinical Frailty Scale, HR: heart rate, SBP: systolic blood pressure, LOC: loss of consciousness, DOAC: direct oral anticoagulant

	OR	95% CI lower	95% CI upper	p-value	Hypothesis
Gender: female	0.9956	0.2345	3.9742	0.995	Do not reject null
Age	1.0239	0.9903	1.0708	0.219	Do not reject null
Transport to ED: private	3.4259	0.8477	14.7518	0.082	Do not reject null
Triage category 2	539794.9425	0		0.993	
Triage category 3	487950.2305	0		0.993	
Triage category 4	639756.9688	0		0.993	
“Lowest GCS”	0.8893	0.6443	1.46	0.503	Do not reject null
Baseline GCS less than 15: yes	3.1071	0.6027	13.3009	0.138	Do not reject null
Documented dementia: yes	2.3377	0.3217	11.23	0.325	Do not reject null
CFS score	1.0946	0.8233	1.4756	0.534	Do not reject null
Lowest HR recorded >100	1	0	1.61388E + 60	1	Do not reject null
Lowest HR recorded 60-100	12245166.78	0		0.994	Do not reject null
Highest SBP recorded 121-160	0.6452	0.1547	3.2741	0.56	Do not reject null
Highest SBP recorded 161-180	0		2.6477E + 64	0.993	
Highest SBP recorded 181-200	0		3.92613E + 82	0.995	
“Dangerous mechanism”: yes	12.5	0.4656	337.0641	0.084	Do not reject null
LOC: yes	0.401	0.0211	2.3485	0.4	Do not reject null
Amnesia: yes	1.5179	0.2132	6.9926	0.622	Do not reject null
Amnesia of events before impact: yes	0		1.64461E + 68	0.994	
Confusion at time of review in ED: yes	1.6042	0.3198	6.5802	0.526	Do not reject null
Headache: yes	0.4762	0.025	2.8094	0.495	Do not reject null
Vomiting >2 episodes: yes	4.0833	0.19	36.4515	0.246	Do not reject null
Suspicion for skull fracture on clinical exam: yes	1.6381	0.2294	7.5922	0.561	Do not reject null
New neurology: yes	12.5	0.4656	337.0641	0.084	Do not reject null
Anticoagulated: DOAC	5.7	1.4	25.1	0.016	Reject null
Anticoagulated: warfarin	0		1.7927E + 206	0.995	
Aspirin or other anti-platelet: aspirin	0.8854	0.0457	5.48	0.912	Do not reject null
Aspirin or other anti-platelet: clopidogrel	0		2.64423E + 91	0.994	

Dangerous mechanisms of injury and new focal neurological deficits demonstrated large effect estimates (both OR 12.5; p = 0.084) but were imprecise, with wide CIs. No significant associations were observed for suspected skull fracture, loss of consciousness, vomiting, or confusion at ED assessment.

## Discussion

In this imaging-selected cohort, CT-positive cases were older (median 80 years, IQR 78-87), and most injuries were low-energy falls, consistent with population data showing high rates of TBI in adults over 75 and a predominance of level-ground falls [41,75,76].

Anticoagulation, particularly with DOACs, was associated with increased odds of intracranial pathology, suggesting it may be a plausible risk marker in this cohort. However, effect sizes should be interpreted cautiously. The multivariable model is likely unstable given only nine CT-positive events, reflected by the wide CIs. A parsimonious or penalized regression approach would be preferable for future analyses.

New focal neurological deficits were more common among CT-positive patients (11.1% vs 1.0%), supporting their potential role as a marker of intracranial injury. Vomiting and suspicion for skull fracture were observed in a minority of cases and were not statistically significant.

The high overall rate of negative CTs in this cohort underscores the potential utility of integrating biomarkers or other risk-stratification tools into future ED protocols to better identify adults at low risk of intracranial pathology. Inclusion of younger adults in our study may dilute associations observed in the older population, limiting the generalizability of the findings to scoring tools designed for older adults. Overall, age, anticoagulation, and new neurological deficits emerge as potential indicators of intracranial pathology that warrant further study in unselected older ED populations.

Implementation considerations

While these findings are hypothesis-generating, they highlight potential opportunities to optimize CT use in older adults presenting after a fall. Documentation of anticoagulation status, baseline cognitive function, and frailty could be systematically incorporated into ED assessment to better identify higher-risk patients.

Enhanced capture of new neurological deficits and standardized injury history may further support risk stratification. However, any modification of imaging protocols should be approached cautiously, as prospective validation in an unselected ED fall population is required before considering CT avoidance or rule modification. These results can inform local workflow and documentation practices but do not provide sufficient evidence to change current imaging standards.

Limitations

Several limitations must be acknowledged. This was a retrospective, single-center study that relied on chart abstraction, limiting precision and generalizability. The small number of CT-positive events (n = 9) reduces statistical power, produces wide CIs, and increases the risk of type II errors. Inclusion of only patients who underwent CT introduces selection bias by excluding clinically cleared mild cases and younger patients. It precludes the estimation of the true sensitivity, specificity, or negative predictive value of any clinical decision rule, which is an essential consideration for studies aiming to reduce unnecessary imaging.

Predictor variables, including head impact, neurological deficits, and frailty, may have been misclassified due to reliance on clinical documentation. Finally, the absence of detailed anticoagulation data, such as dosing, adherence, or laboratory monitoring, further limits the assessment of bleeding risk and constrains the generalizability of these findings beyond our local ED and study timeframe.

Planned further research

We plan to use the included dataset and literature review to inform a prospective analysis of patients ≥65 years presenting with features in keeping with mTBI to investigate predictive factors and develop a reproducible scoring system to safely reduce imaging and length of stay in this cohort.

## Conclusions

Our study identifies anticoagulation, older age, and new neurological deficits as potential indicators of intracranial pathology in adults presenting after a fall. At the same time, vomiting and suspected skull fracture showed weaker, non-significant associations.

The small number of CT-positive cases and the imaging-selected nature of our cohort limit precision and preclude estimation of decision-rule performance or CT avoidance. These findings are therefore hypothesis-generating, highlighting factors that may help prioritize risk assessment in elderly patients. They will conduct a prospective study of an unselected ED fall population and develop a predictive framework to guide safer, more targeted cranial imaging in patients ≥65 years.

## References

[1] Lindner G, Woitok BK (2021). Emergency department overcrowding: analysis and strategies to manage an international phenomenon. Wien Klin Wochenschr.

[2] Sprivulis PC, Da Silva J-A, Jacobs IG, Jelinek GA, Frazer ARL (2006). The association between hospital overcrowding and mortality among patients admitted via Western Australian emergency departments. Med J Aust.

[3] Schneider SM, Gallery ME, Schafermeyer R, Zwemer FL (2003). Emergency department crowding: a point in time. Ann Emerg Med.

[4] Stirparo G, Kacerik E, Andreassi A (2023). Emergency department waiting-time in the post pandemic era: new organizational models, a challenge for the future. Acta Biomed.

[5] Close JC, Lord SR, Antonova EJ (2012). Older people presenting to the emergency department after a fall: a population with substantial recurrent healthcare use. Emerg Med J.

[6] Close J, Ellis M, Hooper R, Glucksman E, Jackson S, Swift C (1999). Prevention of falls in the elderly trial (PROFET): a randomised controlled trial. Lancet.

[7] Tinetti ME, Williams CS (1997). Falls, injuries due to falls, and the risk of admission to a nursing home. N Engl J Med.

[8] Tung M, Sharma R, Hinson JS, Nothelle S, Pannikottu J, Segal JB (2018). Factors associated with imaging overuse in the emergency department: a systematic review. Am J Emerg Med.

[9] Menon DK, Schwab K, Wright DW, Maas AI (2010). Position statement: definition of traumatic brain injury. Arch Phys Med Rehabil.

[10] Maas AIR, Menon DK, Adelson PD (2017). Traumatic brain injury: integrated approaches to improve prevention, clinical care, and research. Lancet Neurol.

[11] Leo P, McCrea M (2011). Epidemiology. Textbook of Traumatic Brain Injury.

[12] Korley FK, Kelen GD, Jones CM, Diaz-Arrastia R (2016). Emergency department evaluation of traumatic brain injury in the United States, 2009-2010. J Head Trauma Rehabil.

[13] de Wit K, Merali Z, Kagoma YK, Mercier É (2020). Incidence of intracranial bleeding in seniors presenting to the emergency department after a fall: a systematic review. Injury.

[14] Vos PE, Alekseenko Y, Battistin L (2012). Mild traumatic brain injury. Eur J Neurol.

[15] Marincowitz C, Lecky FE, Townend W, Borakati A, Fabbri A, Sheldon TA (2018). The risk of deterioration in GCS13-15 patients with traumatic brain injury identified by computed tomography imaging: a systematic review and meta-analysis. J Neurotrauma.

[16] Tourigny JN, Boucher V, Paquet V (2022). External validation of the updated Brain Injury Guidelines for complicated mild traumatic brain injuries: a retrospective cohort study. J Neurosurg.

[17] Cassidy JD, Carroll LJ, Peloso PM (2004). Incidence, risk factors and prevention of mild traumatic brain injury: results of the WHO Collaborating Centre Task Force on mild traumatic brain injury. J Rehabil Med.

[18] Yang LJ, Lassarén P, Londi F (2024). Risk factors for traumatic intracranial hemorrhage in mild traumatic brain injury patients at the emergency department: a systematic review and meta-analysis. Scand J Trauma Resusc Emerg Med.

[19] Fuller G, McClelland G, Lawrence T, Russell W, Lecky F (2016). The diagnostic accuracy of the HITSNS prehospital triage rule for identifying patients with significant traumatic brain injury: a cohort study. Eur J Emerg Med.

[20] Hodgkinson S, Pollit V, Sharpin C, Lecky F (2014). Early management of head injury: summary of updated NICE guidance. BMJ.

[21] Mooney JS, Yates A, Sellar L (2011). Emergency head injury imaging: implementing NICE 2007 in a tertiary neurosciences centre and a busy district general hospital. Emerg Med J.

[22] Stiell IG, Wells GA, Vandemheen K (2001). The Canadian CT head rule for patients with minor head injury. Lancet.

[23] Haydel MJ, Preston CA, Mills TJ, Luber S, Blaudeau E, DeBlieux PM (2000). Indications for computed tomography in patients with minor head injury. N Engl J Med.

[24] Smits M, Dippel DW, de Haan GG (2005). External validation of the Canadian CT Head Rule and the New Orleans Criteria for CT scanning in patients with minor head injury. JAMA.

[25] Stiell IG, Clement CM, Rowe BH (2005). Comparison of the Canadian CT Head Rule and the New Orleans Criteria in patients with minor head injury. JAMA.

[26] Papa L, Stiell IG, Clement CM (2012). Performance of the Canadian CT Head Rule and the New Orleans Criteria for predicting any traumatic intracranial injury on computed tomography in a United States Level I trauma center. Acad Emerg Med.

[27] Mower WR, Hoffman JR, Herbert M, Wolfson AB, Pollack CV Jr, Zucker MI (2005). Developing a decision instrument to guide computed tomographic imaging of blunt head injury patients. J Trauma.

[28] Émond M, Grenier D, Morin J (2017). Emergency department stay associated delirium in older patients. Can Geriatr J.

[29] Jagoda AS, Bazarian JJ, Bruns JJ Jr (2008). Clinical policy: neuroimaging and decisionmaking in adult mild traumatic brain injury in the acute setting. Ann Emerg Med.

[30] (2025). Head injury: assessment and early management. https://www.nice.org.uk/guidance/ng232.

[31] Valente JH, Anderson JD, Paolo WF (2023). Clinical policy: Critical issues in the management of adult patients presenting to the emergency department with mild traumatic brain injury: approved by ACEP board of directors, February 1, 2023 clinical policy endorsed by the Emergency Nurses Association (April 5, 2023). Ann Emerg Med.

[32] NSW Ministry of Health (2017). NSW Health Agency for Clinical Innovation. Initial management of closed head injuries. Sydney (NSW): NSW Health. Adult trauma clinical practice guidelines: initial management of closed head injury in adults.

[33] Trauma Victoria (2014). Traumatic Brain Injury Guideline. In. Traumatic Brain Injury Guideline.

[34] Schachar JL, Zampolin RL, Miller TS, Farinhas JM, Freeman K, Taragin BH (2011). External validation of the New Orleans Criteria (NOC), the Canadian CT Head Rule (CCHR) and the National Emergency X-Radiography Utilization Study II (NEXUS II) for CT scanning in pediatric patients with minor head injury in a non-trauma center. Pediatr Radiol.

[35] Foks KA, van den Brand CL, Lingsma HF (2018). External validation of computed tomography decision rules for minor head injury: prospective, multicentre cohort study in the Netherlands. BMJ.

[36] Alzuhairy AK (2020). Accuracy of Canadian CT head rule and New Orleans criteria for minor head trauma; a systematic review and meta-analysis. Arch Acad Emerg Med.

[37] Davey K, Saul T, Russel G, Wassermann J, Quaas J (2018). Application of the Canadian computed tomography Head rule to patients with minimal head injury. Ann Emerg Med.

[38] van Loveren K, Singla A, Sinvani L (2021). Increased emergency department hallway length of stay is associated with development of delirium. West J Emerg Med.

[39] Béland E, Nadeau A, Carmichael PH (2021). Predictors of delirium in older patients at the emergency department: a prospective multicentre derivation study. CJEM.

[40] Joseph JW, Elhadad N, Mattison ML, Nentwich LM, Levine SA, Marcantonio ER, Kennedy M (2024). Boarding duration in the emergency department and inpatient delirium and severe agitation. JAMA Netw Open.

[41] (2025). Head injuries in Australia 2020-21. https://www.aihw.gov.au/reports/injury/head-injuries-in-australia-2020-21.

[42] Peterson AB KS (2020). Deaths from fall-related traumatic brain injury — United States, 2008-2017. MMWR Morb Mortal Wkly Rep.

[43] Taddei G, Caproni S, Pietrantonio A (2025). Evidence-based indications for repeat head CT after mild traumatic brain injury: a systematic review and meta-analysis. Neurosurg Rev.

[44] Brown AJ, Witham MD, George J (2011). Development of a risk score to guide brain imaging in older patients admitted with falls and confusion. Br J Radiol.

[45] de Wit K, Mercuri M, Clayton N (2023). Derivation of the falls decision rule to exclude intracranial bleeding without head CT in older adults who have fallen. CMAJ.

[46] de Wit K, Parpia S, Varner C (2020). Clinical predictors of intracranial bleeding in older adults who have fallen: a cohort study. J Am Geriatr Soc.

[47] Jeanmonod R, Asher S, Roper J (2019). History and physical exam predictors of intracranial injury in the elderly fall patient: a prospective multicenter study. Am J Emerg Med.

[48] Hamden K, Agresti D, Jeanmonod R, Woods D, Reiter M, Jeanmonod D (2014). Characteristics of elderly fall patients with baseline mental status: high-risk features for intracranial injury. Am J Emerg Med.

[49] Pages PJ, Boncoeur-Martel MP, Dalmay F, Salle H, Caire F, Mounayer C, Rouchaud A (2020). Relevance of emergency head CT scan for fall in the elderly person. J Neuroradiol.

[50] Kudu E, Altun M, Danış F, Karacabey S, Sanri E, Denizbasi A (2025). Validating the falls decision rule: optimizing head CT use in older adults with ground-level falls. CJEM.

[51] Bouvier D, Oris C, Brailova M, Durif J, Sapin V (2020). Interest of blood biomarkers to predict lesions in medical imaging in the context of mild traumatic brain injury. Clin Biochem.

[52] Oris C, Kahouadji S, Durif J, Bouvier D, Sapin V (2023). S100B, actor and biomarker of mild traumatic brain injury. Int J Mol Sci.

[53] Karamian A, Farzaneh H, Khoshnoodi M, Maleki N, Karamian A, Stufflebeam S, Lucke-Wold B (2025). Diagnostic accuracy of S100B in predicting intracranial abnormalities on CT imaging following mild traumatic brain injury: a systematic review and meta-analysis. Neurocrit Care.

[54] Sedaghat F, Notopoulos A (2008). S100 protein family and its application in clinical practice. Hippokratia.

[55] Alexiou ES, Vlachodimitropoulou L, Alexiou GA (2023). S100B as a biomarker in traumatic brain injury. Biomarkers in Trauma, Injury and Critical Care.

[56] Wichmann TO, Babaee A, Duch K (2025). A head-to-head comparison of S100B and GFAP/UCH-L1 for detection of traumatic intracranial lesions in a Scandinavian trauma cohort. Scand J Trauma Resusc Emerg Med.

[57] Calcagnile O, Undén L, Undén J (2012). Clinical validation of S100B use in management of mild head injury. BMC Emerg Med.

[58] Undén L, Calcagnile O, Undén J, Reinstrup P, Bazarian J (2015). Validation of the Scandinavian guidelines for initial management of minimal, mild and moderate traumatic brain injury in adults. BMC Med.

[59] Petzold A, Keir G, Lim D, Smith M, Thompson EJ (2003). Cerebrospinal fluid (CSF) and serum S100B: release and wash-out pattern. Brain Res Bull.

[60] Kleindienst A, Ross Bullock M (2006). A critical analysis of the role of the neurotrophic protein S100B in acute brain injury. J Neurotrauma.

[61] Ferrara M, Bertozzi G, Volonnino G (2022). Glymphatic system a window on TBI pathophysiology: a systematic review. Int J Mol Sci.

[62] Kanner AA, Marchi N, Fazio V (2003). Serum S100beta: a noninvasive marker of blood-brain barrier function and brain lesions. Cancer.

[63] Zimmer DB, Cornwall EH, Landar A, Song W (1995). The S100 protein family: history, function, and expression. Brain Res Bull.

[64] Yardan T, Erenler AK, Baydin A, Aydin K, Cokluk C (2011). Usefulness of S100B protein in neurological disorders. J Pak Med Assoc.

[65] Langeh U, Singh S (2021). Targeting S100B protein as a surrogate biomarker and its role in various neurological disorders. Curr Neuropharmacol.

[66] Oris C, Bouillon-Minois JB, Pinguet J (2021). Predictive performance of blood S100B in the management of patients over 65 years old with mild traumatic brain injury. J Gerontol A Biol Sci Med Sci.

[67] Faisal M, Vedin T, Edelhamre M, Forberg JL (2023). Diagnostic performance of biomarker S100B and guideline adherence in routine care of mild head trauma. Scand J Trauma Resusc Emerg Med.

[68] Jones CM, Harmon C, McCann M, Gunyan H, Bazarian JJ (2020). S100B outperforms clinical decision rules for the identification of intracranial injury on head CT scan after mild traumatic brain injury. Brain Inj.

[69] Kahouadji S, Salamin P, Praz L (2020). S100B blood level determination for early management of ski-related mild traumatic brain injury: a pilot study. Front Neurol.

[70] Oris C, Bouillon-Minois JB, Kahouadji S (2024). S100B vs. "GFAP and UCH-L1" assays in the management of mTBI patients. Clin Chem Lab Med.

[71] Karamian A, Farzaneh H, Khoshnoodi M, Maleki N, Rohatgi S, Ford JN, Romero JM (2025). Accuracy of GFAP and UCH-L1 in predicting brain abnormalities on CT scans after mild traumatic brain injury: a systematic review and meta-analysis. Eur J Trauma Emerg Surg.

[72] Northern Health. Quality Account 2023-24. Melbourne (VIC (2025). Quality account. https://www.nh.org.au/publications/quality-account/.

[73] Sternberg SA, Wershof Schwartz A, Karunananthan S, Bergman H, Mark Clarfield A (2011). The identification of frailty: a systematic literature review. J Am Geriatr Soc.

[74] Patel S, Maria-Rios J, Parikh A, Okorie ON (2023). Diagnosis and management of elevated intracranial pressure in the emergency department. Int J Emerg Med.

[75] Capizzi A, Woo J, Verduzco-Gutierrez M (2020). Traumatic brain injury: an overview of epidemiology, pathophysiology, and medical management. Med Clin North Am.

[76] Centers for Disease Control and Prevention (2025). Surveillance Report of Traumatic Brain Injury-related Emergency
Department Visits, Hospitalizations, and Deaths. Surveillance Report of Traumatic Brain Injury-Related Emergency Department Visits, Hospitalizations, and Deaths.

